# A map of the human neocortex showing the estimated overall myelin content of the individual architectonic areas based on the studies of Adolf Hopf

**DOI:** 10.1007/s00429-016-1228-7

**Published:** 2016-05-02

**Authors:** Rudolf Nieuwenhuys, Cees A. J. Broere

**Affiliations:** 1 The Netherlands Institute for Neuroscience, Royal Netherlands Academy of Arts and Sciences, Meibergdreef 47, 1105 BA Amsterdam, The Netherlands; 2 The Abcoudean Institute of Advanced Study, Papehof 25, 1391 BD Abcoude, The Netherlands

**Keywords:** Architectonics, Cytoarchitectonics, Myeloarchitectonics, Neocortex, Overall myelin content, Neuroimaging

## Abstract

During the period extending from 1910 to 1970, Oscar and Cécile Vogt and their numerous collaborators published a large number of myeloarchitectonic studies on the cortex of the various lobes of the human cerebrum. In a previous publication [Nieuwenhuys et al (Brain Struct Funct 220:2551–2573, 2015; Erratum in Brain Struct Funct 220: 3753–3755, 2015)], we used the data provided by the Vogt–Vogt school for the composition of a myeloarchitectonic map of the entire human neocortex. Because these data were derived from many different brains, a standard brain had to be introduced to which all data available could be transferred. As such the Colin 27 structural scan, aligned to the MNI305 template was selected. The resultant map includes 180 myeloarchitectonic areas, 64 frontal, 30 parietal, 6 insular, 17 occipital and 63 temporal. Here we present a supplementary map in which the overall density of the myelinated fibers in the individual architectonic areas is indicated, based on a meta-analysis of data provided by Adolf Hopf, a prominent collaborator of the Vogts. This map shows that the primary sensory and motor regions are densely myelinated and that, in general, myelination decreases stepwise with the distance from these primary regions. The map also reveals the presence of a number of heavily myelinated formations, situated beyond the primary sensory and motor domains, each consisting of two or more myeloarchitectonic areas. These formations were provisionally designated as the orbitofrontal, intraparietal, posterolateral temporal, and basal temporal dark clusters. Recently published MRI-based in vivo myelin content mappings show, with regard to the primary sensory and motor regions, a striking concordance with our map. As regards the heavily myelinated clusters shown by our map, scrutiny of the current literature revealed that correlates of all of these clusters have been identified in in vivo structural MRI studies and appear to correspond either entirely or largely to known cytoarchitectonic entities. Moreover, functional neuroimaging studies indicate that all of these clusters are involved in vision-related cognitive functions.

## Introduction

The establishment of the relation between particular cortical functions (as determined by neuroimaging techniques) and specific particular cortical structural entities remains a major problem in neurobiology. Currently, these ‘translation’ operations are often provisionally performed by transferring the detected activation loci detected to the three-dimensional version of Brodmann’s famous cytoarchitectural map, produced by Talairach and Tournoux (1988, 1993). However, it has become increasingly clear that this map does not provide sufficient neuroanatomical precision to match the considerable degree of functional segregation suggested by neuroimaging studies (Zilles and Amunts 2010; Geyer et al. 2011; Glasser and van Essen 2011; Amunts and Zilles 2015). A recent finding of great potential significance is that cortical myelin provides excellent MRI contrast, enabling the visualization of structural features relevant for the parcellation of the cortex. Thus, Geyer et al. (2011), using high-resolution MRI, mapped in living subjects the myeloarchitectonic border between the primary somatosensory (S1) and the primary motor cortex (M1), and Glasser and van Essen (2011) demonstrated that regional differences in myelin content across the human cortex can be assessed by mapping the intensity ratio of *T*_1_-weighted and so-called ‘*T*_2_-weighted’ MR images on the cortical surface. Using this approach they identified dozens of features that represent putative areas or areal borders in the living human cortex. Similar findings were reported by other authors (Dick et al. 2012; Waehnert et al. 2014; Tardif et al. 2015; Dinse et al. 2015), opening the perspective of a reliable, structural MRI-based, in vivo myeloarchitectonic parcellation of the human cortex (Turner and Geyer 2014). The findings just mentioned reawakened the interest in the very detailed, but largely forgotten myeloarchitectonic studies on the human cortex of the Vogt–Vogt school, which appeared during the period extending from 1910 to 1970. Recently, one of us (Nieuwenhuys 2013) extensively reviewed these studies. It was concluded that the data available were adequate and sufficient for the composition of a myeloarchitectonic map of the entire human neocortex. Such a map was realized in a subsequent publication (Nieuwenhuys et al. 2015a, b). It includes 180 myeloarchitectonic areas, 64 frontal, 30 parietal, 6 insular, 17 occipital and 63 temporal. The present study is devoted to the creation of a supplementary map in which the estimated overall density of myelinated fibers in the various architectonic areas is indicated. It is based on a meta-analysis of data provided by Adolf Hopf (Hopf 1955, 1956, Hopf and Vitzthum 1957), a prominent collaborator of the Vogts.

## Material

**Fig. 1:**
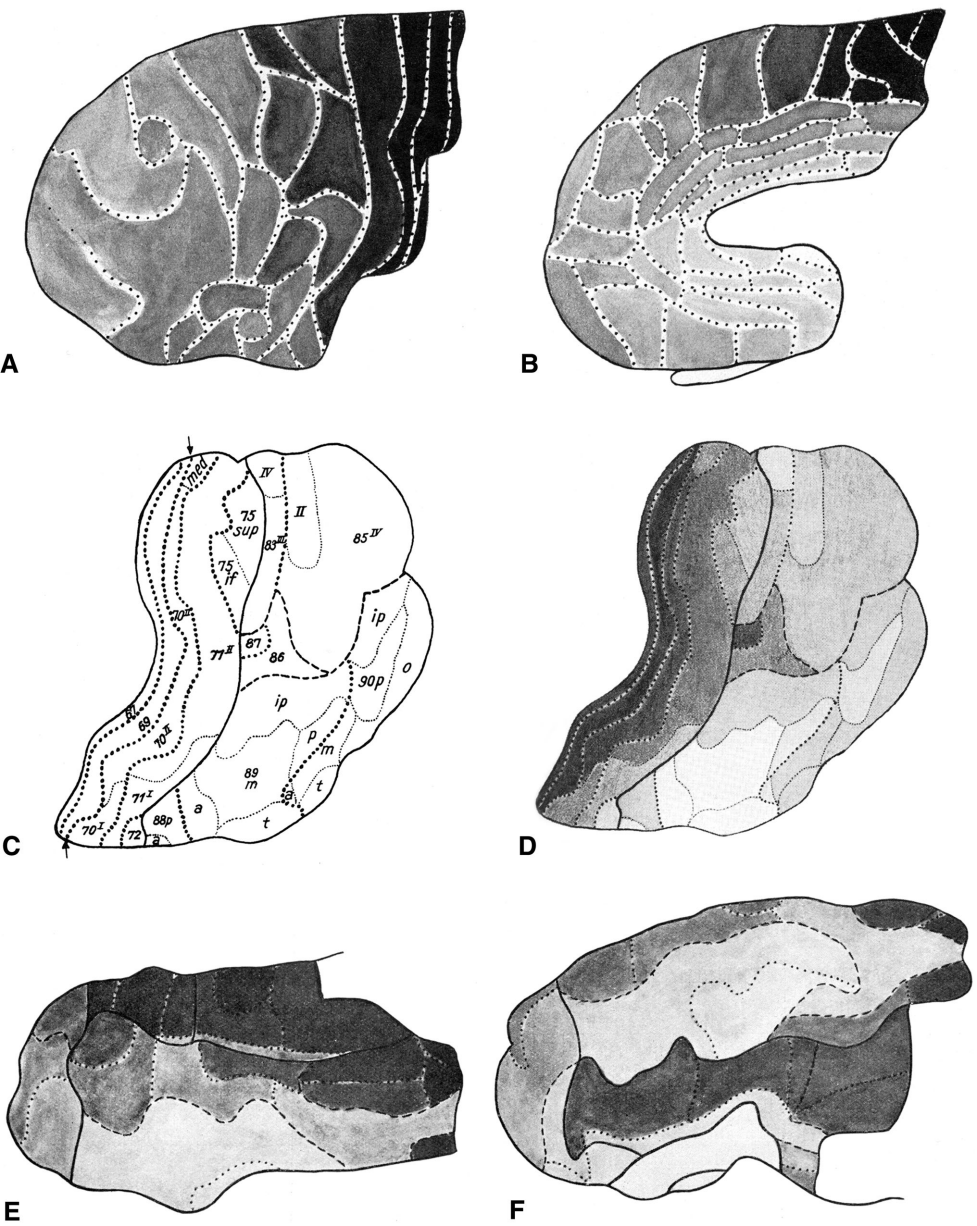
*Maps* showing the overall density of myelinated fibers in the human neocortex. **a**, **b** *lateral* and *medial views* of the frontal lobe (Hopf 1956); **c** The myeloarchitectonic parcellation of the superior surface of the parietal lobe, and **d** the overall myelin content of these areas (Hopf and Vitzthum 1957); **e**, **f** lateral and basal views of the temporal lobe (Hopf 1955). *Gray values* indicate the overall myelin density within each area (*light* *gray* low density, *dark gray* high density)

The material used in this meta-analysis consists of 11 diagrammatic lobar aspect maps, four of the frontal lobe (Hopf (1956), four of the parietal lobe (Hopf and Vitzthum 1957), and three of the temporal lobe (Hopf 1955). In these maps, the overall myelin content of the individual cortical areas is indicated with gray tones of different intensity; dark areas are rich, light ones are poor in myelinated fibers (Fig. 1).

Hopf’s recordings of the myelin content of the various areas were based on the study of serial sections through the various lobes stained using the Weigert method, but details with regard to the transfer of his microscopic observations to the maps are lacking. However, it is known and well documented (Nieuwenhuys 2013; Nieuwenhuys et al. 2015a, 2015b) that the Weigert preparations used by Hopf, and by the Vogt–Vogt school in general, were of exceptionally high quality.

### Procedure

The aim of the procedure is to transfer the myelin density data presented in the 11 diagrammatic lobar aspect maps mentioned above in a reliable and observer-independent way to our new myeloarchiteectonic map of the human neocortex (Nieuwenhuys et al. 2015a, b). The development of this procedure involved the following five steps.

**Fig. 2:**
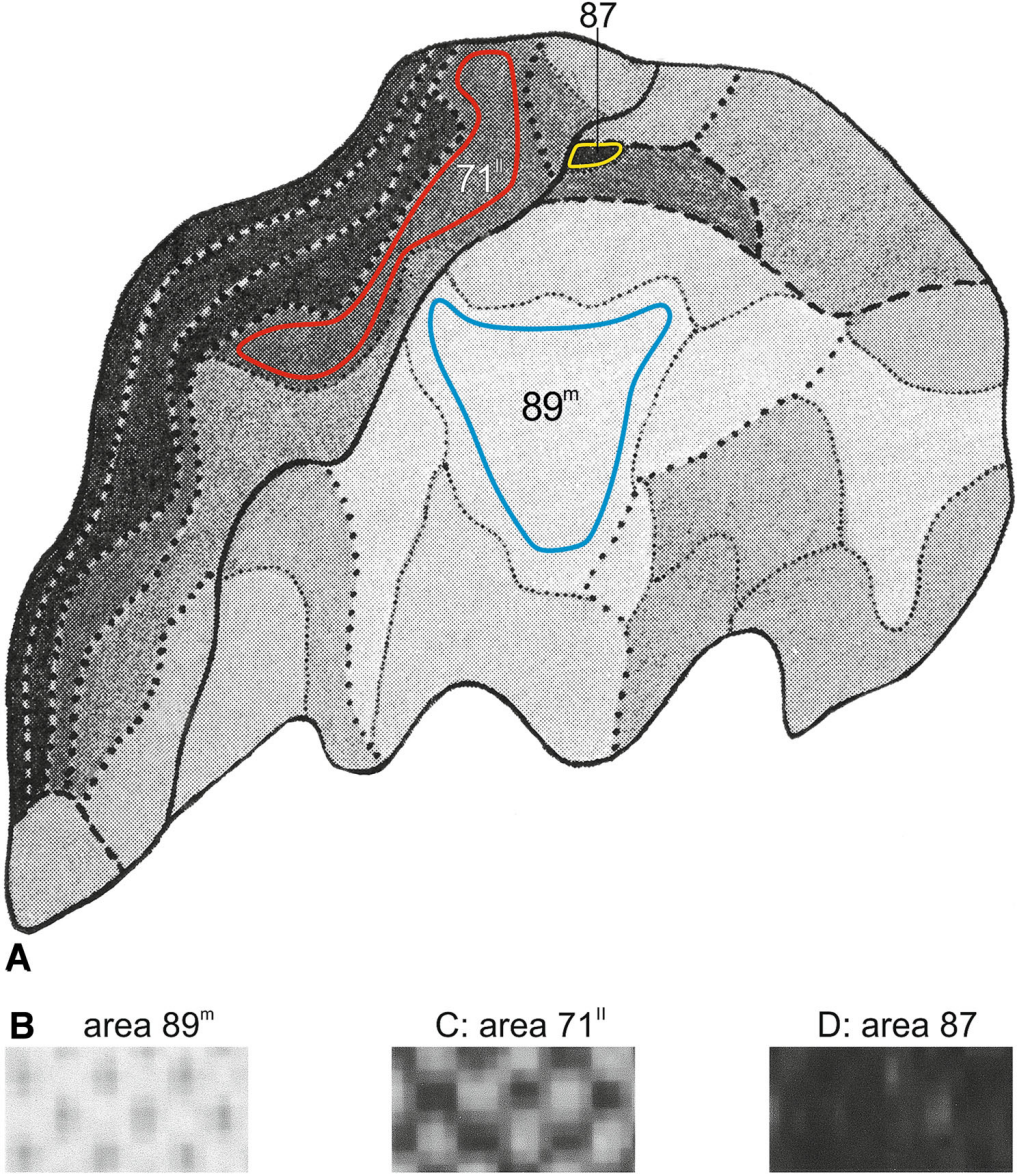
Preparation of the analysis of the myelin density maps. **a** Hopf and Vitzthum’s (1957) map of the* lateral view* of the parietal lobe. The figure was obtained by digitizing the figure from the original publication. In this figure three areas of different intensity shading were selected. In each of these areas an ‘inner contour’ was drawn delineating the field of measurement: blue for Area 89^m^; red for Area 71^II^ and yellow for Area 87. **b**, **c** and **d** show enlargements of a part of these three fields of measurement. The *gray values *of each pixel measured is analyzed using FIGI

1. *Selection of a representative lobar aspect map* As an example, Hopf and Vitzthum (1957) map of the lateral view of the parietal cortex was selected.

2. *Digitalization and determination of pixel gray level values (GLVs) in three representative (sub)areas* Within the lobar aspect map just mentioned, three (sub)areas, viz. the light sub-area 89^m^, the medium dark sub-area 71^II^, and the very dark area 87 were selected for further analysis. Contours were drawn within these (sub)areas which throughout their extent were situated within the pertinent area boundaries (Fig. 2a). The surface areas situated within these ‘inner contours’ were digitized using an Epson Workforce 7525 and FIGI/ImageJ software (ImageJ, Version 2.0.0-rc-39/1.50b; http://imagej.net). The scanning was set at 300 dpi, resulted in approximately 1250 pixels analyzed for area 87, 34000 pixels for Area 7^1I^ and 65000 pixels for Area 89^m^ (Fig. 2b–d). A gray level value (GLV) using FIGI, is attributed to each individual pixel. Pure white has the value of 255 and black the value of 0.

The gray level values of all pixels located within the three inner contours were recorded. The mean of these gray levels (MGL) was determined, and the statistics (extreme values, mean value and SD) calculated. This procedure was carried out five times for each of the three (sub)areas selected (Table 1).

**Table 1:** Conversion of Hopf’s shading technique to mean gray level index

Cortical area	Measurement #	Surface area (pixels)	MGL	GL (min)	GL (max)
Measurements					
Area 87					
	1	1223	54.377	26	142
	2	1357	53.923	26	147
	3	1054	55.156	29	142
	4	1233	55.735	26	142
	5	1413	55.870	26	142
Area 71^II^					
	6	32512	117.832	37	211
	7	31931	118.253	37	210
	8	34268	117.702	42	210
	9	34579	118.331	37	211
	10	36691	118.582	37	210
Area 89^m^					
	11	68018	207.825	125	239
	12	71278	207.745	74	239
	13	61343	207.966	122	239
	14	85104	207.704	122	239
	15	40734	208.177	129	239
Statistics					
Area 87					
	Mean	1256	54.612	26.6	143
	SD	138	0.47	1.32	2.24
Area 71^II^					
	Mean	33960.2	118.14	38	210
	SD	1880	0.364	2.2	0.55
Area 89^m^					
	Mean	65295.4	207.884	114.4	239
	SD	162.36	0.19	22	0

Table 1 gives data of three independent calculations of the MGL using individual measurements of the GL. Statistics show for five measurements of the MGL the SD of the mean of these values <0.01. The MGL value appears not to be influenced by the measurement surface area

This analysis showed the standard deviations of the MGL to be smaller than 0.5 % of the MGL value for each area measured, indicating the determination of the MGL to be reproducible. Hence it is sufficient to perform the procedure of measuring the gray values of the pixels falling within a given area only once.

3. *Determination of MGL for all cortical areas analyzed by Hopf* The MGL of all of the (sub)areas present in each of the eleven lobar aspect maps were determined. The data, thus, collected for the frontal, parietal and temporal lobes are presented in Tables 2, 3, 4.

**Table 2:** Frontal Lobe Densitometry

Field number	Lateral view gray level	Superior view gray level	Medial view gray level	Inferior view gray level	Mean gray level
1			168	134	**151**
2			159		**159**
3			173		**173**
4			159	159	**159**
5				144	**144**
6			168	169	**169**
8				156	**156**
9				147	**147**
10			185		**185**
11			186	196	**191**
12			187		**187**
13			199		**199**
14			215		**215**
15			195		**195**
16			199		**199**
17			190		**190**
18			181		**181**
19			177		**177**
20			181		**181**
21			192		**192**
22			157		**157**
23			162		**162**
24			157		**157**
25			189		**189**
26			159		**159**
27			149		**149**
28			165		**165**
30			145		**145**
31			131		**131**
32			137		**137**
33			160		**160**
34		149	151		**150**
35			144		**144**
36	68	102	108		**93**
37	81	66	89		**77**
38	56	57	50		**54**
39	46	48	43		**46**
40	56	57			**56**
41	125			168	**143**
42	43	47	41		**44**
43	46				**46**
44	74	76			**75**
45	127	109			**118**
46	125	109			**118**
47	134	132	141		**136**
48	148	126	156		**143**
49	163	145	168		**159**
50		147	161		**154**
51	156	147	143	140	**149**
52				149	**149**
53	137	146		155	**146**
54	118	140			**129**
55	89	120			**105**
56	97	106		178	**127**
57	144				**144**
58	118				**118**
59	124			151	**138**
60				87	**87**
61				94	**94**
62				136	**136**
63				141	**141**
64				152	**152**
65				117	**117**
66				123	**123**

**Table 3:** Parietal lobe densitometry

Field number	Dorsal view gray level	Medial view gray level	Lateral view gray level	Parietal operculum gray level	Mean gray level
67	79		88		**84**
67^III^		62			**62**
67^IV^		105			**105**
68^I^				196	**196**
68^II^				189	**189**
68^III^			187	189	**188**
69	98	58	85		**88**
70^med^	136	134			**135**
70^I^	146		122		**138**
70^II^	76		68		**72**
71^m.^		159			**159**
71^I^	159		157		**158**
71^II^	103	109	126		**116**
72	192		186	177	**184**
73^I^				176	**176**
73^II^				164	**164**
73^III^				191	**191**
74^I^				190	**190**
74^II^				198	**198**
75^med^		165			**165**
75^sup^	168				**168**
75^if^	145				**145**
76^s^		191			**191**
76^i^		202			**202**
77		198			**198**
78		201			**201**
79		197			**197**
80		195			**195**
81		174			**174**
82		193			**193**
83^I^		168			**168**
83^II^		197			**197**
83^III^			182		**182**
83^IV^	189	172			**181**
84		170			**170**
85^I^		166			**186**
85^II^	178	188			**183**
85^III^		172			**172**
85^IV^	169		172		**171**
86	129		125		**127**
87	72		56		**64**
88^a^	200		192		**196**
88^p^	181		179		**181**
89^a^	207		204		**206**
89^m^	207		208		**208**
89^p^	207		206		**207**
89^ip^	179		200		**190**
89^t^	209		199		**204**
90^a^	195		206		**201**
90^m^	182		179		**181**
90^p^	190		204		**197**
90^ip^	174		185		**180**
90^t^	182		175		**179**
90^o^	177		178		**178**
91		159			**159**
92		104			**104**
93		113			**113**
94		172			**172**
95		167			**167**
96		159			**159**

**Table 4:** Temporal lobe densitometry

Field number	Lateral view gray level	Superior view gray level	Median view gray level	Inferior view gray level	Mean gray level
120		200		189	**195**
121		196			**196**
122		204			**204**
123		206	196	196	**200**
124			189	189	**189**
125		189			**189**
126		180			**180**
127	165		164	164	**164**
128	149				**149**
129	109	149			**129**
130		170			**170**
131		203			**203**
132		203			**203**
133		218			**218**
134		218			**218**
135		218			**218**
136	81	131			**106**
137	81	102			**92**
138	90	111			**101**
139	150				**150**
140		147			**147**
141		172			**172**
142		169			**169**
143		189			**189**
144		211			**211**
145		92			**92**
146		65			**65**
147		87			**87**
148		65			**65**
149		84			**84**
150		95			**95**
151		65			**65**
152		87			**87**
153		93			**93**
154		92			**92**
155		93			**93**
156		88			**88**
157		72			**72**
158	149				**149**
159	93				**93**
160	93	185			**139**
161	82	96			**89**
162	105				**105**
163	146			120	**133**
164	167			139	**153**
165	110				**110**
166	181			196	**189**
167	204		197	198	**200**
168	205		204	204	**204**
169	100			94	**97**
170	91				**91**
171	75			81	**78**
172	75				**75**
173			89	136	**113**
174	80			89	**85**
175			102	102	**102**
176			103	104	**104**
177			112	113	**113**
178				197	**197**
179			116	117	**117**
180			110	111	**111**
181			186	186	**186**
182			143	143	**143**

4. *Determination of a single MGL for each cortical field* Many of the cortical areas are represented in two or even three or four of Hopf’s lobar aspect maps. Thus, area 53 is present in the medial and inferior aspect maps of the frontal lobe, sub-area 85^IV^ is present in the lateral, superior and medial aspect maps of the parietal lobe, and the apical frontal area 51 is even present in all four of the aspect maps of that lobe. Hopf indicated the overall myelin content of all of the areas in all of his lobar aspect maps with gray tones of different intensity, and we measured the respective MGLs of the gray shading all of these areas. The results of these measurements are included in Tables 2, 3, 4, in which the data derived from the various lobar aspect maps (lateral, superior, medial and inferior) are recorded separately. As a consequence, for many areas two or three, and in the case of area 51 even four gray values are available. In the great majority of cases in which two or more MGLs for a given area were available, the differences between these values were less than 5 % of the MGLs involved. In these cases, we felt justified to take as a mean of the MGLs their arithmetic mean. In case of larger differences in MGL of the same area in different aspect maps of Hopf, a surface area-weighted mean was used. The weight of each MGL of each area used was the value of the surface of the area viewed, divided by the sum of all surface areas viewed of the cortical field analyzed. Alternatively put, in the case of a cortical field present in more than one projection, the surface of the area viewed may change due to a different angle of view. If the MGL values in these different views are widely apart and the surface areas of the different views of the area differ more than 5 % in value, this effect is corrected at calculating the mean MGL. This is done by correcting for the contribution of the values of the MGLs of the different views to the mean of these MGLs by their surface area relative to the total surface area of all views.

5. *Transfer of the MGLs to our myeloarchitectonic map* The MGL, as determined for the various areas, was taken as the gray value for the printing of the areas in our myeloarchitectonic map (Nieuwenhuys et al. 2015a, b). This transfer could be realized because the parcellations of the temporal, frontal and parietal cortices used by Hopf (1955, 1956) and Hopf and Vitzthum (1957), respectively, correspond directly and completely to those indicated in our myeloarchitectonic map.

### The new map

**Fig. 3:**
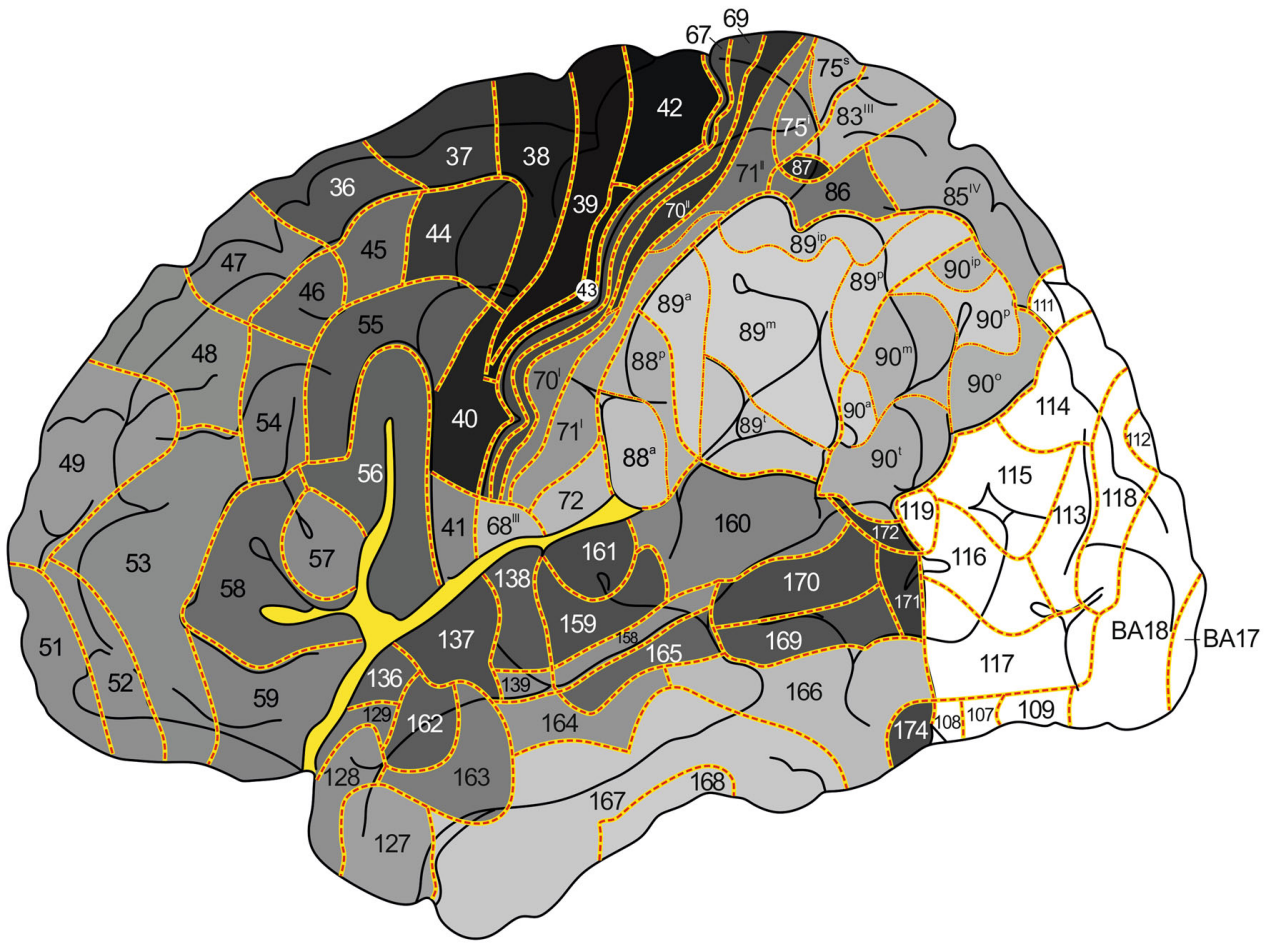
*Map* showing the overall density of myelinated fibers in the human neocortex; lateral aspect

**Fig. 4:**
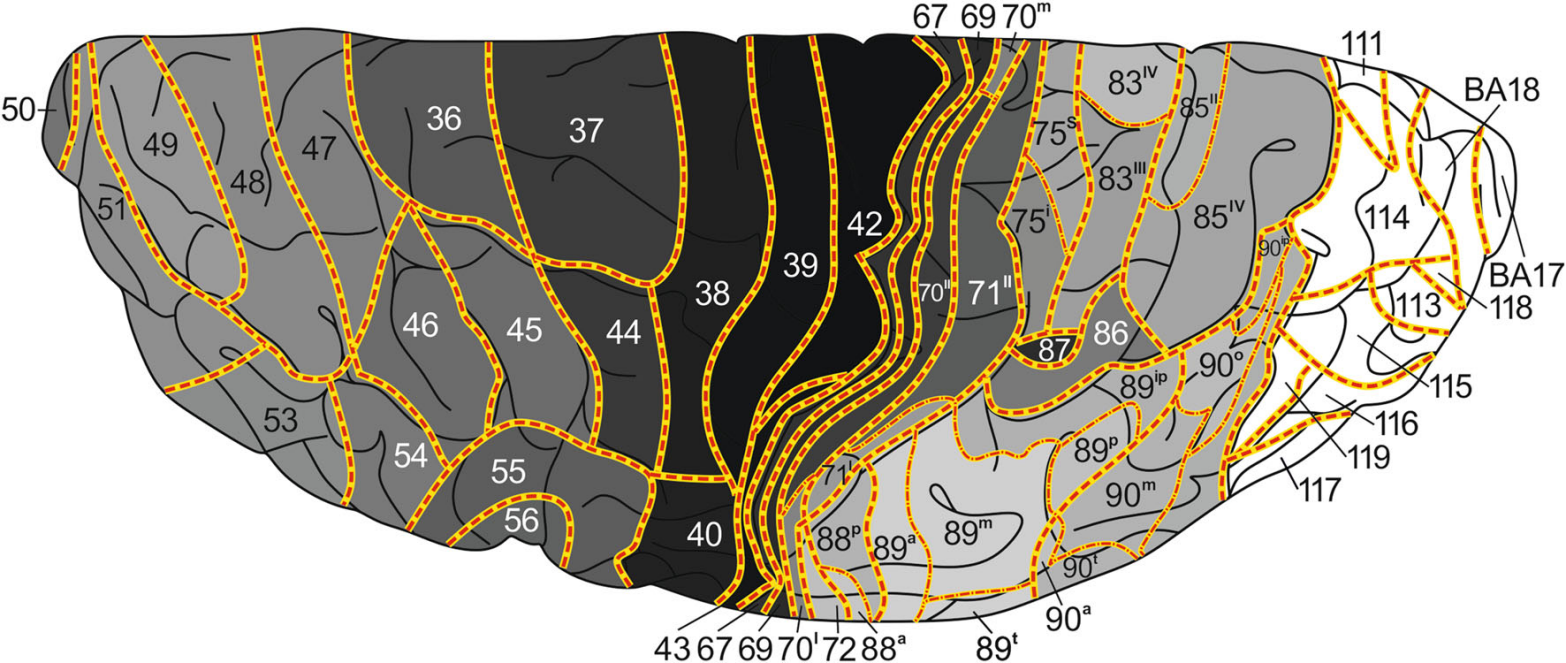
*Map* showing the overall density of myelinated fibers in the human neocortex; superior aspect

**Fig. 5:**
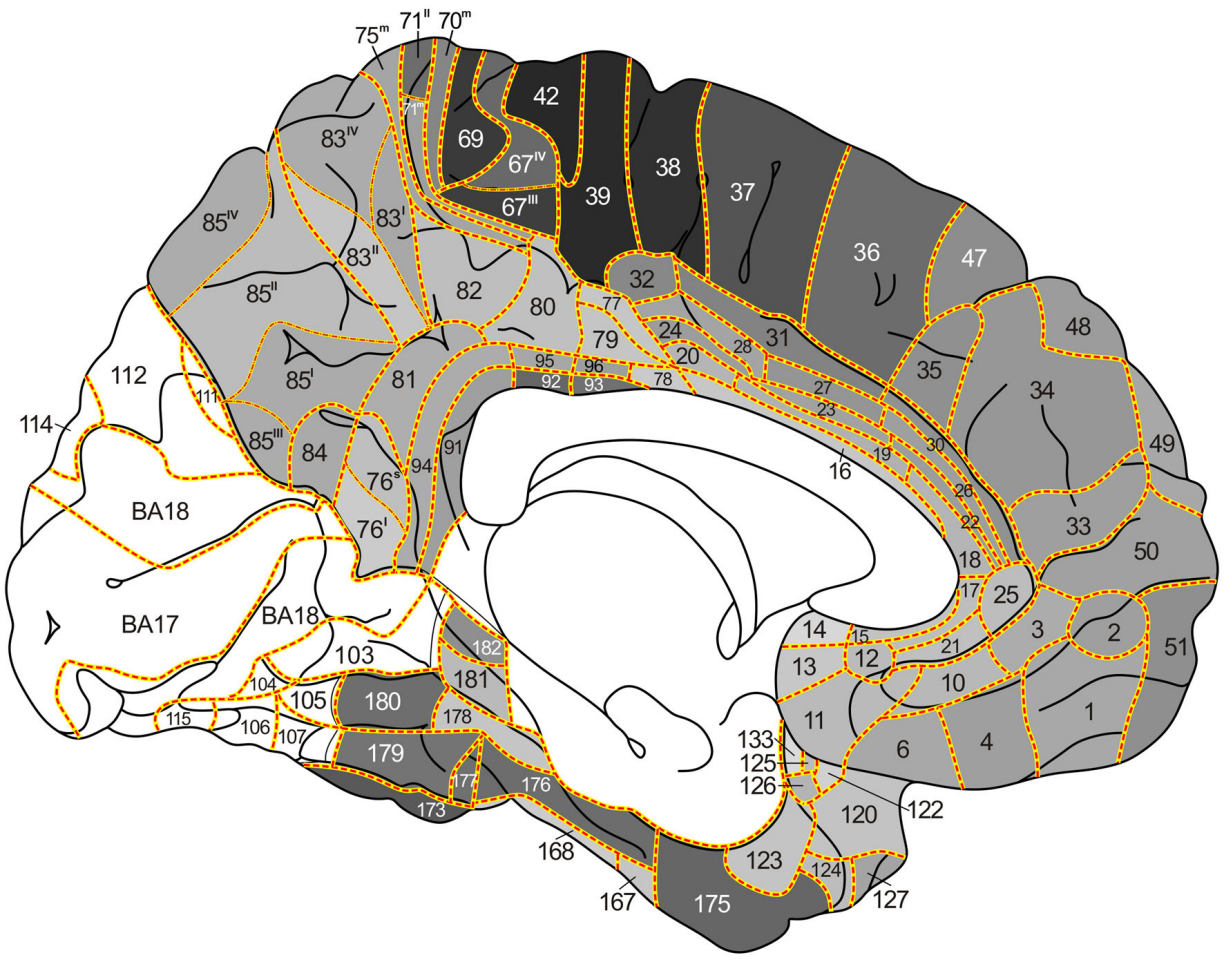
*Map* showing the overall density of myelinated fibers in the human neocortex; medial aspect

**Fig. 6:**
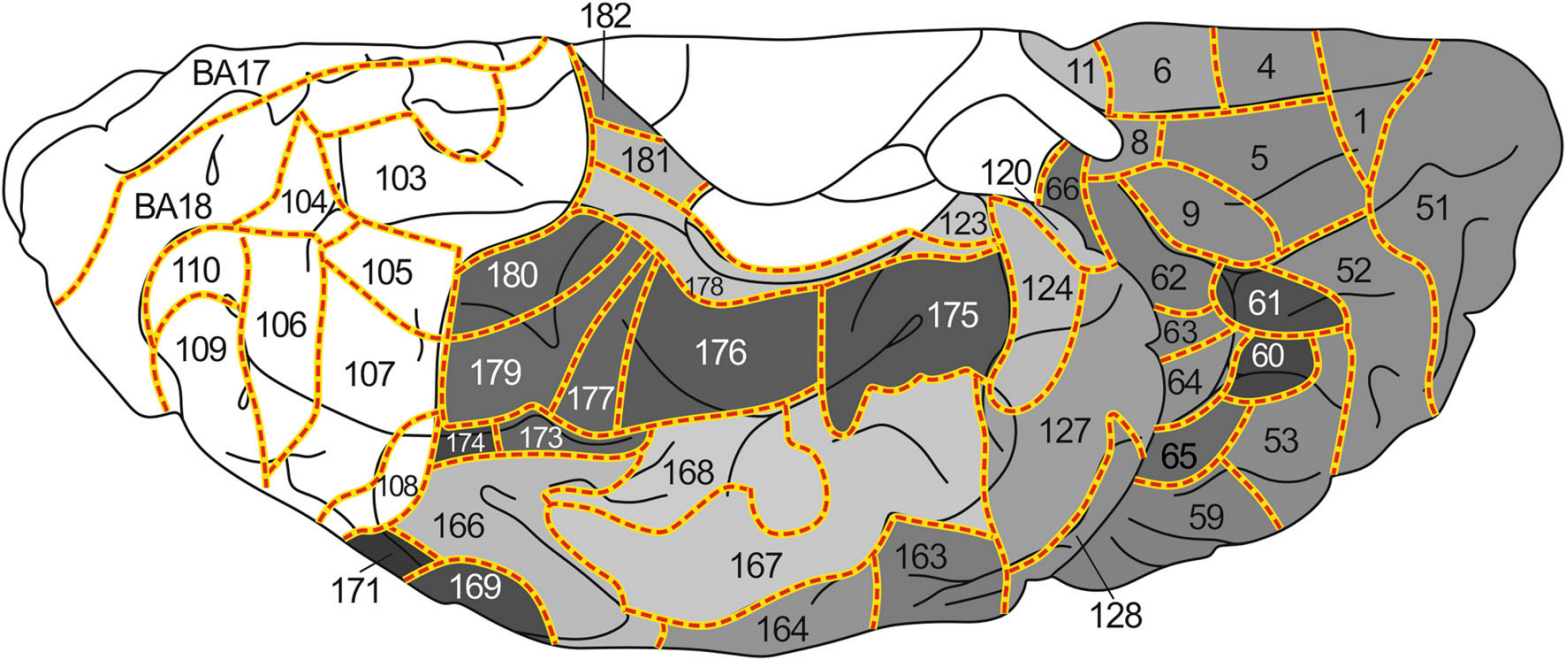
*Map* showing the overall density of myelinated fibers in the human neocortex; basal aspect

**Fig. 7:**
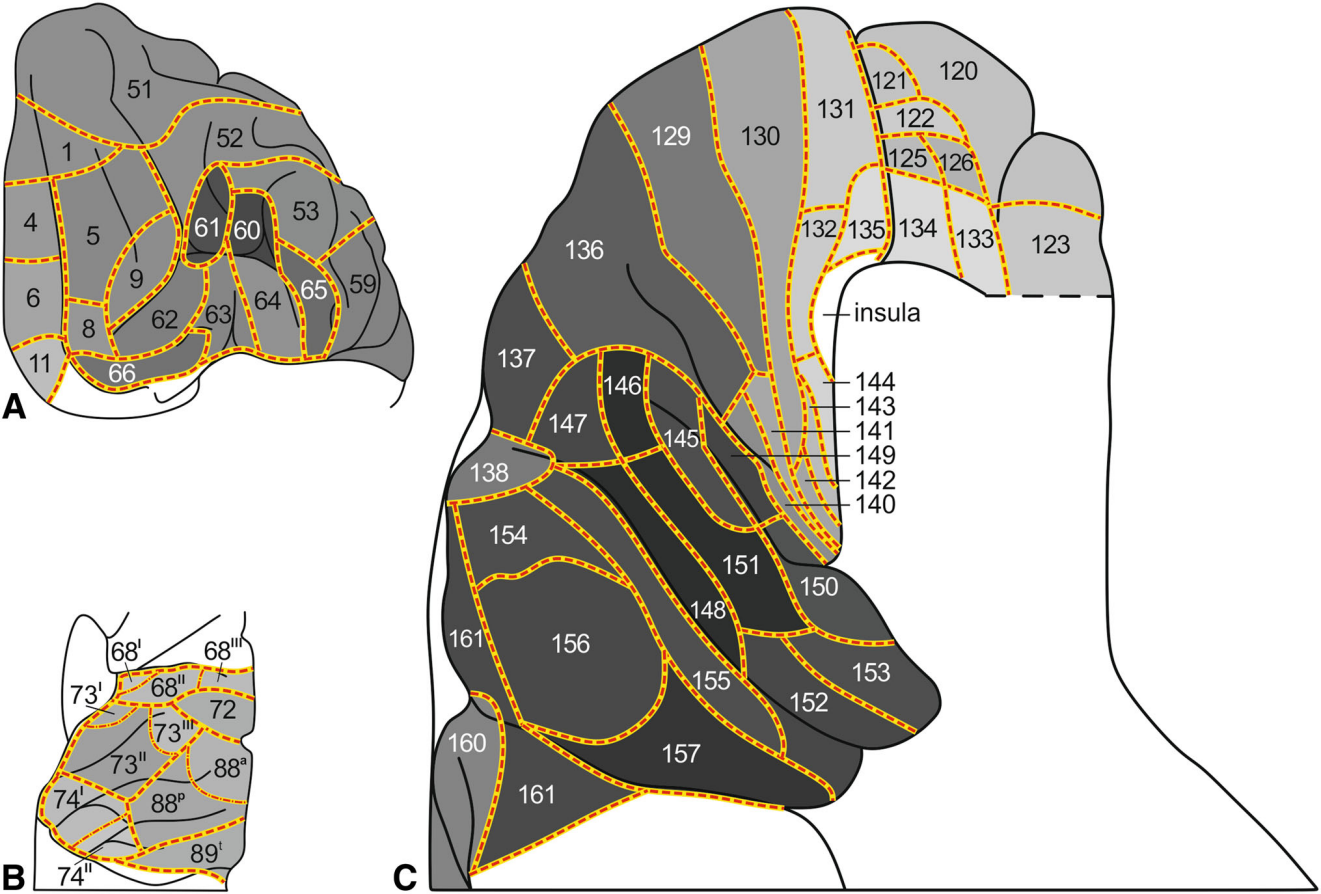
Details of certain parts of our map showing the overall density of myelinated fibers in the human neocortex: **a** orbitofrontal cortex; **b** parietal operculum; **c** supratemporal plane. Note that the latter is depicted twice as large as the remaining two parts

The aim of the present study is the creation of a map of the human neocortex showing the overall estimated density of the myelinatad fibers in the various architectonic areas, based on the histological studies of Hopf (Hopf 1955, 1956; Hopf and Vitzthum 1957). This map is aimed to serve as a reference for the numerous recent MRI-based in vivo studies of the myeloarchitecture of the human cortex. In a previous publication (Nieuwenhuys et al. 2015a), we presented a myeloarchitectonic map of the human neocortex based on histological data provided by the Vogts and their numerous collaborators. Because these data are derived from many different brains, a standard brain had to be introduced to which all of the data available could be transferred. As such, the Colin 27 structural scan, aligned to the MNI 305 template was selected. The resultant map includes 180 myeloarchitectonic areas, 64 frontal, 30 parietal, 6 insular, 17 occipital and 63 temporal. The designation of the various areas with simple Arabic numerals, introduced by Oscar Vogt (1910, 1911) for the frontal and parietal cortices, has been extended over the entire neocortex. It is of note that the numerals used in our maps have nothing to do with those used by Brodmann (1909) (also Arabic) for his cytorchitectonic areas. The ‘myelin density map’ presented here (Figs. 3, 4, 5, 6, 7) is, as already mentioned, based on histological data provided by Hopf (1955, 1956) and Hopf and Vitzthum (1957). Because the areal subdivision of the cortex employed by these authors is identical to that of the Vogt–Vogt school (of which they were members themselves), their myelin density data could be directly transferred to our standard map. However, in the parietal cortex Hopf and Vitzthum (1957) divided many of the 30 ‘Vogt–Vogt areas’ into two or more (up to six) sub-areas. They specified these subareas by adding Roman numerals or abbreviated positional designations (a for anterior, m for medial etc.) to the Arabic numerals indicating the various areas (Fig. 1c). This sub-areal parcellation of the parietal cortex has been included in our myelin density maps (Figs. 3, 4, 5, 7b).

### Limitations of the new map

Our new map shows the following important limitations:

1. It is incomplete, because myelin density data on the insular and occipital cortices are not available.
2. The map shows only the exposed, and not the intrasulcal parts of the various areas. This is a serious limitation because in the human almost two-thirds of the cortex are hidden in the depths of the sulci. However, the frontal areas 43 and the parietal areas 67 and 69, which are actually hidden in the central sulcus, are exposed as narrow strips in Figs. 3 and 4.
3. The map does not yield any information on the interhemispheric and interindividual variability of the various myeloarchitectonic areas. This is another serious limitation because this variability is known to be considerable for numerous areas (Geyer 2013).

### Features shown by the new map

[For the localization of the various anatomical structures (lobes, lobuli, gyri, sulci), mentioned in this and the next section, we refer to the atlas of the standard brain included in Nieuwenhuys et al. (2015a)].

1. The map shows first and foremost that the myeloarchitectonic areas of the frontal, parietal and temporal lobes show considerable differences in their overall myelin content.
2. Vogt and Vogt (1919) indicated that the architecture of the cerebral cortex shows *gradations*, i.e, discontinuous, stepwise changes of architectonic features. The studies of Hopf (1955, 1956) and Hopf and Vitzthum (1957), on which our map is based, have shown that such steps manifest themselves clearly in the areal differences of the apparent density of the myelinated cortical fibers. In general it may be said that the areas which receive the large sensory projections are heavily myelinated, and that myelination decreases with the distance from these areas. This can be observed in the lateral parts of the parietal lobe, proceeding posteriorly from the heavily myelinated primary somatosensory cortex (areas 67, 69–71^II^), in the postcentral gyrus, to the superior parietal lobule (sub-areas 75^I^, 75^s^ 83^III^, 85^IV^; Fig. 4), or to the inferior parietal lobule (sub-areas 88^p^, 89^a^, 89^m^; Fig. 3), and in the medial parietal lobe if we pass from the paracentral lobule to the precuneus (sub-areas 75^m^, 83^I^, 83^II^, 85^II^; Fig. 5). The primary auditory cortex, which covers the anterior and posterior transverse temporal gyri of Heschl (areas 145–157), is densely myelinated just like the primary somatosensory cortex (Fig. 7c). Distinct stepwise decreases in the content of myelinated fibers can be observed proceeding anteromedially (areas 136, 129, 130, 131; Fig. 7c), or anterolaterally (areas 161, 159, 139, 158; Figs. 3, 7c) from this primary sensory cortex. A similar gradation can be observed in the occipital lobe, in which the area striata [Brodmanns’ cytoarchitectonic area (BA) 17] and the adjacent area occipitalis (BA 18) are more densely myelinated than the area praeoccipitalis (BA 19) (Hopf 1955).
  Evident stepwise decreases in myelination can also be observed in the frontal lobe if we pass from the densely myelinated primary motor cortex (areas 39, 42, 43) to the frontal pole, and from the medial frontal cortex (areas 33–38), via the anterior cingulate gyrus, to the corpus callosum (Figs. 3, 4, 5). In the parietal lobe, however, the myelination increases if we pass from the anteromedial areas 79 and 80, via areas 95 and 96, to the pericallosal areas 92 and 93.
3. The human neocortex contains, apart from the primary sensory and motor areas, several other densely myelinated formations, each consisting of two or more myeloarchitectonic areas. We designate these formations provisionally as the orbitofrontal, intraparietal, posterolateral temporal, and basal temporal dark clusters.
  The *orbitofrontal dark cluster* comprises the areas 60 and 61 (Figs. 6, 7a).
  The *intraparietal dark cluster* is named so because it is situated in and around the anterior part of the intraparietal sulcus. It consists of the very dark area 87 (GLV 86) and the somewhat lighter area 86 (GLV 127) (Figs. 3, 4).
  The *posterolateral temporal dark cluster* occupies the posterior part of the middle temporal gyrus. It comprises the very dark areas 171 (GLV 78) and 172 (GLV 75) and the slightly lighter areas 169 and 170 (GLVs 97 and 91, respectively) (Fig. 3).
  The *basal temporal dark cluster* consists of areas 173-177 and 179–180, and occupies most of the anterior three quarters of the lateral occipitotemporal or fusiform gyrus (Figs. 5, 6). It is surrounded by a belt of lightly myelinated areas,which includes the medially situated areas 128 and 123, the rostral area 124, and the more laterally situated areas 166–168 (Figs. 3, 5, 6).

## Discussion

In this section, some features shown by our map will be compared with the results of in vivo myelin content mappings and related structural and functional data.

In the MRI-based in vivo maps of cortical myelin content, published by Glasser and Van Essen (2011), Geyer (2013), Lutti et al. (2014), Mangeat et al. (2015), Tardif et al. (2015), Dinse et al. (2015) and Waehnert et al. (2016), the heavily myelinated somatosensory, auditory, visual and primary motor cortices are clearly discernable from surrounding less myelinated regions.

The *orbitofrontal dark cluster* (Fig. 7a) corresponds to a small heavily myelinated area visible in two of the myelin-based in vivo maps produced by Glasser and Van Essen (2011: Fig. 7a, e), as well as to a small face-responsive area observed by Rajimehr et al. (2009: Fig. 3a). The myeloarchitectonic areas 60 and 61, which together form the orbitofrontal dark cluster (Fig. 7a) correspond presumably to the cytoarchitectonic sub-areas 47^1^ and 47^2^, distinguished by Sarkissov et al. (1955), as well as to area 47/12 m of Öngür et al. (2003).

The banks of the intraparietal sulcus are known to be occupied by a series of anteroposteriorly arranged multimodal association areas (Culham and Kanwisher 2001; Grefkes and Fink 2005). In these areas impulses supplied by the dorsal visual processing stream are correlated with stimuli derived from other sensory modalities and transferred to various parts of the premotor cortex. Prominent among these centers are the anterior intraparietal area (AIP), which is located on the lateral bank of the most anterior part of the intraparietal sulcus, and the slightly more posteriorly situated ventral intraparietal area (VIP). Choi et al. (2006), who studied the cortex surrounding the anterior part of the intraparietal sulcus, delineated two distinct cytoarchitectonic areas in this region, which they designated as the human intraparietal areas 1 and 2 (hip 1, 2). These cytoarchitectonic areas appeared to correspond with the functionally defined areas AIP and VIP, respectively. The cytoarchitectonic areas hip 1 and hip 2 probably correspond to the myeloarchitectonic areas 87 and 86, which form together *the intraparietal dark cluster* (Figs. 3, 4). This cluster is clearly visible in the myelin-based in vivo maps produced by Glasser and Van Essen (2011: Fig. 3a), Sereno et al. 2013: Fig. 4) and Mangeat et al. 2015: Fig. 3a). It coincides with myelogenetic area 17 of Flechsig (1920).

Our discussion of the *posterolateral temporal dark cluster*, which comprises areas 169–172, will be preceded by a brief excursion to the occipital lobe. This lobe was not included in Hopf’s mapping program of the overall myelin content of the various cortical areas. Hence it is left white in our maps (Figs. 3, 4, 5, 6). The myeloarchitecture of the preoccipital region (BA 19) of this lobe was, however, thoroughly analyzed by Lungwitz (1937). He delineated 17 areas within this region, which he designated with combinations of two-to-four letters. We transferred these fields to our maps and indicated them with the numbers 103–119 (Nieuwenhuys et al. 2015a; Figs. 3–6).

Functional imaging studies have shown that the most rostral part of the convex lateral surface of the occipital lobe is occupied by an area which is strongly and specifically activated by moving visual stimuli (Watson et al. 1993; Huk et al. 2002; Walters et al. 2003). This functional area is known as the middle temporal visual area (MT) or V5. Morphologically it is characterized by its dense myelination (Clarke and Miklossy 1990; Tootell and Taylor 1995). According to Watson et al. (1993) it corresponds with the, also heavily myelinated myelogenetic area 16 of Flechsig (1920). Malikovic et al. (2007) found that MT has a cytoarchitectonic equivalent, which they designated as hOc5.

If we plot the results of the morphological and fMRI studies just mentioned on our map (Fig. 3), it appears that MT/V5 occupies a territory encompassing area 119 and the rostral parts of areas 116 and 117. One would expect that in in vivo MRI studies of the myelin content of the human cerebral cortex, the heavily myelinated posterolateral temporal dark cluster, and the also heavily myelinated occipital area MT/V5, would form together a single continuum; however, in the in vivo myelin content mappings of Sereno et al. (2013: Figs. 2, 4), Glasser et al. (2014: Figs. 1, 3) and Mangeat et al. (2015: Fig. 11c), heavy myelination is clearly confined to an occipital region corresponding with the area MT/V5 as observed in histological and fMRI studies. Glasser and Van Essen (2011: Fig. 8a, f) observed a moderately heavily myelinated “finger” extending forward from the densely myelinated occipital pole. Although this formation shows some resemblance to our posterolateral dark cluster (Fig. 3), Glasser and Van Essen emphasized that this “finger” is situated within the occipital lobe, and corresponds positionally to the cytoarchitectonic hOc5 area. Kolster et al. (2010), using fMRI and retinotopic mapping techniques, delineated in the human brain a motion-sensitive complex comprising four different retinotopically organized areas, a superior and inferior occipital one, and a superior and inferior temporal one. The superior occipital area appeared to correspond to MT/V5. The inferior occipital area was designated as pV4t, standing for putative V4 transitional zone. The superior and inferior temporal areas were designated as pMSTv (putative ventral part of the middle superior temporal area) and pFST (putative fundus of the superior temporal area), respectively. In a subsequent paper, devoted to correspondences between retinotopic areas and in vivo myelin maps in the human visual cortex, Abdollahi et al. (2014) reported that the heavily myelinated spot discussed above does not coincide with MT, but rather involves a considerable portion of pMSTv, or is situated directly antero-superiorly to that area. This implies (cf. Abdollahi et al. 2014, Figs. 8a, 9, 10) that this heavily myelinated spot corresponds positionally at least in part with the posterolateral temporal dark cluster in our map. However, it is of note that the region just discussed is highly and variably folded, and that there is a considerable variability in the relationships between architectonic areas and folds. These features hamper the registration and interpretation of the various areas involved.

The *basal temporal dark cluster*, finally, consists as already mentioned of areas 173–177 and 179–180 (Fig. 6), and occupies most of the anterior three quarters of the fusiform gyrus; the posterior quarter of this gyrus forms part of the occipital lobe. Functional imaging studies (Sergent et al. 1992; Kanwisher et al. 1997) have shown that the cortex of the fusiform gyrus is involved in the discrimination of faces. According to the fMRI study of Rajimehr et al. (2009: Fig. 3a), this so-called fusiform face area (FFA) extends throughout the length of the fusiform gyrus. The myelin-based in vivo maps produced by Glasser and Van Essen (2011: Fig. 10a, d) show that a strip of cortex that is more heavily myelinated than the cortex on either side, extends anteriorly from the basal occipital cortex into the temporal part of the fusiform gyrus. Glasser and Van Essen (2011) note that this strip extends less far anteriorly than the FFA as determined by Rajimehr et al. (2009), and than a heavily myelinated formation observed by Hopf (1955), which corresponds with the basal temporal dark cluster of the present meta-analysis. However, they consider it likely that this discrepancy is due to the technical limitations of their approach. The anterior portion of the basal temporal dark cluster, which comprises the areas 175 and 176 of our map (Fig. 6), clearly corresponds with the cytoarchitectonic area 20tc of Sarkissov et al. (1955); the posterior part of this cluster, which includes the areas 173, 174, 177, 179 and 180 of our map, roughly corresponds with the cytoarchitectonic areas FG3 and FG4, recently described by Lorenz et al. (2015).

If we survey the data on the four heavily myelinated clusters just discussed, it appears that all of them have been identified in in vivo structural MRI studies, and correspond either entirely or largely to known cytoarchitectonic entities. Moreover, all of these clusters have been shown to be involved in vision-related cognitive functions.

We are currently working on a mesh for our published myeloarchitectonic map (Nieuwenhuys et al. 2015a, b), and we intend to add a mesh for the myelin density map included in the present paper.

Finally, it should be noted that the studies of Hopf (1955, 1956; Hopf and Vitzthum 1957), forming the basis of the present meta-analysis, are not confined to the overall myelin content of the various cortical areas. They also present systematic analyses of the distribution of other myeloarchitectonic features, including the caliber of the fibers in the cortical radial bundles, the extent of the band of Kaes-Bechterew, and the relation of the bands of Baillarger to each other and to neighboring layers. Recent advances in MRI analysis of cortical microstructure (Aggarwal et al. 2015; Weiskopf et al. 2015; Waehnert et al. 2016) render it possible to visualize salient features of the myeloarchitectonic organization of the cortex. It may be expected that the detailed and systematic studies of Hopf will play a prominent role in the interpretation of these new in vivo results.
